# Correction: Enhanced environmental remediation through hybrid adsorption-photocatalysis using ZnO/TiO_2_-CaAlg composite catalysts

**DOI:** 10.1039/d6ra90027e

**Published:** 2026-03-13

**Authors:** Nesrine Ammouchi, Abdallah Zaiter, Atmane Djermoune, Nawal Bouzenad, Nada Hamrouche, Amdjed Abdennouri, Youghourta Belhocine, Najoua Sbei, Seyfeddine Rahali, Tarek H. Taha, Fehmi Boufahja, Walid Elfalleh, Stefania Garzoli, Hamdi Bendif, Djihane Slimane Ben Ali

**Affiliations:** a Laboratory for Research on the Physico-Chemistry of Surfaces and Interfaces (LRPCSI), Department of Sciences and Technology, Faculty of Technology, University 20 August 1955 Skikda 21000 Algeria; b Laboratory of Applied Chemistry and Materials Technology, Larbi Ben M'Hidi-Oum El Bouaghi University Oum El Bouaghi 04000 Algeria; c Scientific and Technical Research Center in Physical and Chemical Analyses BP 384 Bou-Ismail RP 42004 Tipaza Algeria; d Laboratory of Catalysis, Bioprocesses and Environment (LCBE), University 20 August 1955 Skikda 21000 Algeria; e Department of Organic Chemistry, University of Alcalá 28871 Alcalá de Henares Madrid Spain; f Department of Chemistry, College of Science, Qassim University Buraydah Saudi Arabia; g Department of Biology, College of Science, Imam Mohammad Ibn Saud Islamic University (IMSIU) Riyadh 11623 Saudi Arabia hlbendif@imamu.edu.sa; h Department of Chemistry and Technologies of Drug, Sapienza University P. le Aldo Moro 5,00185 Rome Italy stefania.garzoli@uniroma1.it; i Department of Petrochemical, Faculty of Technology, University of 20 Août1955 El Hadaiek Road, B. O. 26 21000 Skikda Algeria dj.slimanebenali@univ-skikda.dz

## Abstract

Correction for ‘Enhanced environmental remediation through hybrid adsorption-photocatalysis using ZnO/TiO_2_-CaAlg composite catalysts’ by Nesrine Ammouchi *et al.*, *RSC Adv.*, 2026, **16**, 8226–8242, https://doi.org/10.1039/D5RA09465H.

The authors regret that Stefania Garzoli was incorrectly listed as a corresponding author. The correct corresponding authors are Hamdi Bendif & Djihane Slimane Ben Ali, as shown above.

The Royal Society of Chemistry apologises for these errors and any consequent inconvenience to authors and readers.

